# Diagnostic accuracy of OGUS, Southend halo score and halo count in giant cell arteritis

**DOI:** 10.3389/fmed.2024.1320076

**Published:** 2024-01-26

**Authors:** Edoardo Conticini, Paolo Falsetti, Suhel Gabriele Al Khayyat, Silvia Grazzini, Caterina Baldi, Francesca Bellisai, Stefano Gentileschi, Marco Bardelli, Claudia Fabiani, Luca Cantarini, Bhaskar Dasgupta, Bruno Frediani

**Affiliations:** ^1^Rheumatology Unit, Department of Medicine, Surgery and Neurosciences, University of Siena, Siena, Italy; ^2^Ophthalmology Unit, Department of Medicine, Surgery and Neurosciences, University of Siena, Siena, Italy; ^3^Rheumatology Department, Mid and South Essex University Hospitals NHS Foundation Trust, Southend University Hospital, Essex, United Kingdom

**Keywords:** ultrasonography, vasculitis, giant cell arteritis, Horton arteritis, diagnosis

## Abstract

**Objectives:**

Ultrasound has a paramount role in the diagnostic assessment of giant cell arteritis (GCA); Southend halo score (HS), halo count (HC), and OMERACT GCA Ultrasonography Score (OGUS) are the first quantitative scores proposed in this setting. The aim of this study was therefore to assess the diagnostic accuracy of these scores in a real-life scenario, as well as to evaluate their optimal cutoff, also with respect to disease extent, sex, and age.

**Methods:**

We retrospectively collected clinical, serological, and US findings of all patients referred for the first time to our vasculitis clinic in the suspicion of GCA.

**Results:**

A total of 79 patients were included, and a definite diagnosis of GCA was made in 43 patients. For OGUS, the ROC curve showed an optimal cut point of 0.81 (sensitivity 79.07% and specificity 97.22%). For HC and HS, the optimal cutoff values were > 1.5 (sensitivity 76.7% and specificity 97.2%) and > 14.5 (sensitivity 74.4% and specificity 97.2%), respectively. No relevant differences were assessed when patients were stratified according to disease extent, age, and sex. Compression sign (CS) was positive in 34 of 38 patients with cranial GCA and negative in all controls and LV-GCA.

**Conclusion:**

All three scores display good sensitivity and excellent specificity, although the cutoff was slightly different than proposed. In particular, for OGUS, a threshold of 0.81 could be employed for diagnostic purposes, although it was developed solely for monitoring. Due to its high sensitivity and specificity, CS should be always assessed in all patients referred with a suspicion of cranial GCA.

## Highlights

- Southend halo score (HS) and halo count (HC) have a good diagnostic accuracy, which is not influenced by disease extent, age, and sex.
- Even though the OMERACT GCA Ultrasonography Score (OGUS) was developed for disease monitoring, our study suggests its potential diagnostic role.
- Compression sign (CS) has the highest sensitivity and specificity for cranial GCA: Temporal arteries US should always comprise a dynamic evaluation.

## Introduction

Giant cell arteritis (GCA) is a large-vessel vasculitis affecting the aorta and its major branches. Due to the high morbidity arising from irreversible, organ-threatening complications, an early diagnosis and prompt adequate treatment are mandatory. In this regard, in contradistinction to the temporal artery (TA) biopsy advocated in ACR 1990 criteria (1), imaging has a paramount role in the diagnosis and assessment of GCA. An ultrasound (US) of TA and axillary (AxA) arteries, due to its wide availability, rapidity, and lack of ionizing radiation, is most employed for both large vessels (LV) and cranial GCA.

Nevertheless, despite growing evidence supporting its routine use for diagnosis (2) and follow-up (3–6), US has several shortcomings in clinical practice: first the poor training of specialists facing the first symptoms of GCA, including rheumatologists; second, the paucity of studies that include also the LV-GCA phenotype in addition to the more common cranial one (7); third, the lack of validated quantitative scores, which limits its use for clinical trials (8) and multicenter studies.

The first quantitative score reports using the Southend Halo score (HS) (9) found an association with male sex, disease activity, ocular ischemia, and intimal hyperplasia on temporal artery biopsy. Thereafter, the Outcome Measures in Rheumatology (OMERACT) ultrasonography large-vessel vasculitis working group, after defining and testing elementary lesions in GCA (10, 11), has recently developed a novel, provisional score for disease monitoring (12). Both the HS and OMERACT GCA Ultrasonography Score (OGUS) displayed an excellent agreement and proved to be sensitive to changes during follow-up as well as to correlate with markers of inflammation and Birmingham Vasculitis Activity Score (BVAS) in one study (13).

Thus, we aimed to evaluate the quantitative halo scores (9, 12) in a real-life setting in order to assess their diagnostic accuracy and feasibility.

The primary endpoint of the study was a retrospective assessment of the specificity and sensitivity of OGUS, as well as to determine its optimal cutoff values, in a cohort of patients referred to our clinic with suspected GCA.

Secondary endpoints were to retrospectively assess the accuracy of the halo scores with respect to disease extent (LV and cranial) and to compare it with semiquantitative and quantitative scores already employed in our clinical practice.

## Materials and methods

### Study population

We retrospectively collected clinical, serological, and US findings of all patients referred to Vasculitis Clinic, Rheumatology Unit, University Hospital of Siena, in the suspicion of GCA from January 2020 to January 2023.

Patients could be referred by other clinicians or through our fast-track pathway, in which patients suffering from sudden visual impairment and/or other symptoms of GCA and an increase in erythrocyte sedimentation rate (ESR) and/or C-reactive protein (CRP) were immediately referred to our clinic.

Inclusion criteria were the availability of the following: a minimum core set of blood examinations (14), including hemoglobin (Hb), ESR, and CRP; US findings, including intima–media thickness (IMT), compressibility, and the presence of “halo sign” in AxA and common temporal, parietal, and frontal branches of both TA; a definite clinical diagnosis, which was performed by a single rheumatologist experienced in vasculitis and expressed as follows: cranial GCA, LV-GCA, cranial and LV-GCA, and no GCA.

Exclusion criteria were the unavailability of the abovementioned findings and a previous diagnosis of GCA in remission at the time of the assessment, as well as concomitant or previous treatment with anti-IL6 agents.

### Ultrasonography

US examination was carried out by two rheumatologists experienced in US employing an Esaote MyLab X8, equipped with two linear (4–15 and 18–22 MHz) probes, and an Esaote MyLab Twice, equipped with two linear (4–13 and 6–18 MHz) probes. The vessels assessed were AxA and common temporal, parietal, and frontal branches of TA, the latter being evaluated only with high-frequency probes. Color Doppler frequency was set at 9–12.3 MHz and pulse repetition frequency at 2–3 KHz, while gain was adjusted at just below the threshold of artifacts. The burden of vascular inflammation was measured through IMT and scored using halo count (HC), HS (9), and OGUS (12). IMT measurements were manually performed evaluating the thickness from the luminal–intimal interface to the medial–adventitial one, in a longitudinal scan during systole and reported in millimeters (15, 16). The occurrence of low compressibility of any branch of TA was also recorded.

### Statistical analysis

A binomial regression analysis was performed to obtain diagnostic cutoffs of different components of OGUS (total, LV, cranial, etc.). Various ROC curves were calculated comparing the halo score components with the diagnosis of GCA as the gold standard.

### Ethics

This study was conducted in accordance with the Declaration of Helsinki and its late amendments and approved by the local ethics committee (Rhelabus, protocol number 22271).

## Results

A total of 79 subjects were evaluated with suspicion of GCA, and a clinical diagnosis was made in 43 of them (mean age 76.42 years; 24 women): 29 patients suffered from cranial GCA, 9 presented with involvement of both cranial and extracranial arteries, while 5 had only LV-GCA. Clinical and serological features of the patients, including PET findings, are reported in Table 1.

**Table 1:** Clinical and serological features of GCA patients.

		GCA patients (*n* = 43)
Sex		24 women, 19 men
Mean age ± SD		76.42 ± 7.44 years
GCA subtype (*N*, %)	Cranial	29, 67%
	Cranial + LV	9, 21%
	LV	5, 12%
CRP (mean ± SD)		7.52 ± 5.74 mg/dL
ESR (mean ± SD)		69.28 ± 38.9 mm/h
Hb (mean ± SD)		11.08 ± 0.92 g/L
Ocular symptoms (*N*, %)		18, 41.9%
Headache (*N*, %)		19, 44.2%
Scalp tenderness (*N*, %)		12, 28%
Jaw claudication (*N*, %)		12, 28%
PMR (*N*, %)		10, 23.2%
B symptoms (*N*, %)		13, 30.2%
Relapsing patients (*N*, %)		8, 18.6%
Disease duration of relapsing patients (mean)		48 months
Treatment at the time of the first assessment (*N*, %)		6, 13.9%
	Prednisone (*N*, %)	6, 13.9%
	Methotrexate (*N*, %)	3, 6.9%
TA biopsy		0
PET (*N*, %)		6, 13.9%
**PET uptake**		
	Axillary and subclavian arteries	4, 66.6%
	Thoracic aorta	6, 100%
	Abdominal aorta	4, 66.6%
	Iliac arteries	2, 33.3%
**US findings**		
	**IMT** (mean values expressed in mm) ± SD	
	Right axillary artery	0.93 ± 0.4
	Left axillary artery	0.88 ± 0.34
	Right temporal artery: common branch	0.43 ± 0.19
	Left temporal artery: common branch	0.43 ± 0.21
	Right temporal artery: parietal branch	0.26 ± 0.19
	Left temporal artery: parietal branch	0.23 ± 0.12
	Right temporal artery: frontal branch	0.26 ± 0.16
	Left temporal artery: frontal branch	0.26 ± 0.16
	**HC** (mean ± SD)	2.86 ± 1.8
	**HS** (mean ± SD)	19.9 ± 7.42
	**OGUS** (mean ± SD)	1.01 ± 0.24
	**CS**+	34/38 cranial ± LV-GCA

CRP, C-reactive protein; CS, compression sign; ESR, erythrocyte sedimentation rate; GCA, giant cell arteritis; Hb, hemoglobin; HC, Halo count; HS, Halo score; LV, large vessels; OGUS, OMERACT GCA US Score; PET, positron emission tomography; PMR, polymyalgia rheumatica; SD, standard deviation; TA, temporal artery; US, ultrasonography.

No patient underwent TA biopsy, while PET was requested in 6, in which an involvement of large vessels was suspected: In all of them, imaging displayed a pathological uptake (Meller scale 3) in the territory of the aorta and/or iliac vessels.

The area under the ROC curve (AUROC) for OGUS was 0.980 (95% confidence interval: 0.9534, 1). The ROC curve showed an optimal cut point of 0.81, with a sensitivity of 79.07%, a specificity of 97.22%, and a likelihood ratio (LR) of 28.47. Similar cutoffs were found also when patients were stratified according to disease extent (Table 2), while slightly lower values, although with 100% sensitivity, were reported when our cohort was subdivided for age and sex (Table 3).

**Table 2:** OGUS, stratification for GCA type.

	Cutoff	AUROC	Sensitivity (%) *CI*	Specificity (%) *CI*	LR	*p* value
All GCA	0.81	0.980 *0*.*95–1%*	79 *64*.*8–88*.*6%*	97.2 *85*.*8–99*.*8%*	28.47	<0.0001
Cranial	0.82	0.981 *0*.*95–1%*	79.3 *61*.*1–90*.*1%*	97.2 *85*.*8–99*.*8%*	28.5	<0.0001
LV	0.81	0.972 *0*.*92 – 1%*	80 *37*.*5–98*.*7%*	97.2 *85*.*8–99*.*8%*	28.8	0.0007
Cranial + LV	0.83	0.979 *0*.*98–1%*	77.7 *45*.*5–96%*	97.2 *85*.*8–99*.*8%*	24	<0.0001

AUROC, area under the ROC curve; CI, confidence interval; GCA, giant cell arteritis; LR, likelihood ratio; LV, large vessels; Sens, sensitivity; Spec, specificity.

**Table 3:** OGUS, stratification for sex and age.

	Cutoff	Sensitivity (%)	Specificity (%)	LR	*p* value
Male	>0.78	89.4	91.6	10.7	<0.0001
Female	>0.72	100	95.8	24	
Age ≥ 76	0.7	100	92.8	14	
Age < 76	0.73	100	92.3	13	

LR, likelihood ratio; Sens, sensitivity; Spec, specificity.

For HC, AUROC was 0.979, with a CI of 0.944 to 1 and a *p* < 0.0001. The optimal cutoff value was set at >1.5 with a sensitivity of 76.7% (CI: 62.2–86.8%), a specificity of 97.2% (CI: 85.8–99.8%), and an LR of 27.63. A lower, although high, LR (17) was assessed with a cutoff >0.5 (sensitivity 100%, specificity 94.44%). The same cutoff (>1.5) was found to be the optimal one also for cranial, LV, and LV + cranial GCA, but the latter had the lowest sensitivity (66.6%), while the highest (100%) was found for cranial (Table 4). As for OGUS, these findings were not influenced by sex or age (Table 5). Finally, a positive, statistically significant correlation was found between HC and OGUS (Pearson’s r: 0.841, *p* < 0.001).

**Table 4:** Halo count, stratification for GCA type.

	Cutoff	AUROC	Sensitivity (%) *IC*	Specificity (%) *IC*	LR	*p* value
All GCA	>1.5	0.979 *0*.*94–1%*	76.6 *62*.*2–86*.*8%*	97.2 *85*.*8–99*.*8%*	27.63	<0.0001
Cranial	>1.5	0.980 *0*.*94–1%*	75.8 *57*.*8–87*.*7%*	97.2 *85*.*8–99*.*8%*	27.3	<0.0001
LV	>1.5	0.972 *0*.*92 – 1%*	100 *56*.*5–100%*	97.2 *85*.*8–99*.*8%*	36	0.0007
Cranial + LV	>1.5	0.979 *0*.*94–1%*	66.6 *35*.*4% – 87* 0.*9%*	97.2 *85*.*8–99*.*8%*	24	<0.0001

**Table 5:** Halo count, stratification for sex and age.

	Cutoff	Sensitivity (%)	Specificity (%)	LR	*p* value
Male	≥1	100	91	12	<0.0001
Female	≥1	100	95	24	<0.0001
Age ≥ 76	≥1	100	100		
Age < 76	≥1	100	92	13	

For HS, a cutoff of >14.5 (AUROC: 0.95, sensitivity: 74.4%, specificity: 97.2, and LR: 26.7) was found for all GCA patients, but a lower sensitivity (65.5%) was found for cranial ones (Table 6). In contrast to HC and OGUS, a different optimal cutoff was evidenced for men, in whom an HS >8.5 was associated with 100% sensitivity and 83.3% specificity (Table 7).

**Table 6:** Halo score, stratification for GCA type.

	Cutoff	AUROC	Sensitivity (%) *IC*	Specificity (%) *IC*	LR	*p* value
All GCA	>14.5	0.950 *0*.*89–1%*	74.4 *59*.*7–85%*	97.2 *85*.*8–99*.*8%*	26.7	<0.0001
Cranial	>14.5	0.931 *0*.*82–1%*	65.5 *45*.*3–80%*	97.2 *85*.*8–99*.*8%*	23.5	<0.0001
Cranial + LV	15	0.986 *0*.*95–1%*	88.8 *56*.*5–99*.*4%*	97.2 *85*.*8–99*.*8%*	32	<0.0001

**Table 7:** Halo score, stratification for sex and age.

	Cutoff	Sensitivity (%)	Specificity (%)	LR	*p* value
Male	>8.5	100	83.3	6	<0.0001
Female	>14.5	70.8	95.8	17	<0.0001
Age ≥ 76	>9	95.2	92.3	13	
Age < 76	>9.5	85	92.3	11.1	

Compression signs were positive in 34 of 38 patients with cranial and cranial + LV-GCA, while all controls and LV-GCA displayed negative CS (89% sensitivity and 100% specificity).

## Discussion

The retrospective application of three different US scores to patients with suspected GCA allows for the first time a direct comparison of these methodologies. While some US scores and cutoff values have been proposed, their application is *de facto* restricted to the cohorts in which they were originally applied (9) and few other ones (6, 18). At the same time, OGUS, specifically designed for clinical trials and disease monitoring, is a consensus-based algorithm and has not been applied yet in clinical practice, except for assessing its sensitivity to change after treatment (13, 17), which appeared comparable to HS and HC, although only for TA.

In our sample population, comprising 79 subjects referred to in the suspicion of GCA, we evidenced positive US findings in the majority of patients who eventually were diagnosed with vasculitis, thus confirming the crucial role of US in its diagnostic work-up. Such findings were not influenced by age nor differed between first diagnosis and relapse; the only relevant difference was according to sex and disease extent, as the only GCA patient in whom US was negative had an exclusive involvement of the aorta. This is not surprising, because men have greater IMT than women (9), and at the same time, some vascular territories (i.e., aorta and iliac arteries) cannot adequately be detected by US and require different imaging procedures, such as PET, MRI, and CT. On the other hand, no patient with a final diagnosis of cranial GCA had a fully negative US.

When separately analyzing the three scores taken for examination, an overall good diagnostic accuracy was assessed, although with cutoffs slightly different than proposed.

In particular, OGUS had the best diagnostic performance at a threshold of 0.81, instead of 1.01: The latter resulted in an excellent specificity (100%) but a poor sensitivity (39.53%), while our cutoff displayed a slightly lower specificity (94.44%) but a significantly higher sensitivity (79.07%) with an LR of 28.47.

This finding was predictable, as OGUS was designed for clinical trials and research and not for being employed in a clinical setting nor for diagnostic purposes: In this context, a lower specificity, thus potentially leading to overtreatment of a patient with suspected vasculitis, should be preferred to a 100% specificity with a poor sensitivity, which in real life may lead to a hazardous and harmful undertreatment of a GCA.

On the other hand, our data confirm the excellent specificity of OGUS, applied for the first time in a real-life cohort, and strongly support its use in drug research and trials, in which the need to exclude mimickers is prevalent. Moreover, even though OGUS was developed only for disease monitoring, our study seems to suggest its potential diagnostic role.

For HC, our findings did not substantially differ from the cutoffs previously proposed: an HC ≥2 provided a 76.74% sensitivity and 97.22% specificity, with an LR of 27.63, which are values *de facto* comparable to the ones reported by Molina-Collada et al. (18) for an HC > 1 (sensitivity 80%; specificity 95%) and by van der Geest et al. (9) for an HC ≥ 2 in case of TAB positivity (sensitivity 85% and specificity 70%). On the other hand, despite a similar sensitivity (78%), van der Geest et al. (9) reported a much lower specificity (55%), for an optimal cutoff of 1. Curiously, a specificity comparable to ours (95%) was reported only for a cutoff of 6, 3-fold higher than the optimal one calculated in our cohort.

Such discrepancies are not easy to explain but are potentially due to the occurrence of a high HC in two non-GCA patients from the Southend cohort, which differed from ours in terms of F:M ratio (2.86 vs. 1.26).

On the other hand, it is noteworthy that all three cohorts evidenced a similar sensitivity, despite the differences existing among the three populations: the one by van der Geest et al. (9) and ours double the Spanish one (18) and include predominantly GCA patients, while in the latter, the controls are two thirds of the total. Moreover, and more importantly, we included cranial, LV, and cranial plus LV-GCA; the Southend cohort had only subjects with cranial vasculitis (headache was complained in up to 96% of subjects) and focused on the ischemic hazard, while, conversely, only 5 patients from the study by Molina-Collada et al. (18) fulfilled 1990 ACR criteria. Finally, at the time of the US assessment, four patients were relapsing, while both previous studies included only subjects referred for the first time.

That has a paramount importance in clinical terms, which is the ground of this study: Indeed, despite the application of this score in cohorts composed of different patients, comparable only for sex and age, HC presents the same good sensitivity, also for low or very low cutoffs. This confirms the potential application of HC in daily clinical practice, in which the prevention of ischemic complications prevails over the need to minimize the immunosuppressive treatment. In summary, it is not necessary to reach an HC ≥ 6, which can be assessed only in a minority of patients, to reasonably start glucocorticoids in a patient with suspected GCA.

More relevant discrepancies were conversely evidenced for HS: Lower values resulted in very poor specificity, particularly when compared with the cohort by Molina-Collada et al. (18), who reported a sensitivity and specificity of 86.7 and 95.3%, respectively, for HS ≥ 2.

Conversely, our results found an optimal cutoff of 15, displaying an excellent specificity (97.22%) and a good sensitivity (74.22%), far higher than the one (21%) reported by van der Geest et al. (9) for an HC ≥ 10.

Such a difference is not easy to explain but can be presumably determined by the inclusion of relapsing patients in our cohort, therefore presenting a higher IMT of AxA, and by the higher numbers of LV-GCA. A lower HS can be considered prudentially.

However, regardless of the difference existing for the optimal cutoff, which may also be due to the heterogeneity of the patients included in the studies, both HC and HS, as well as OGUS, proved to be reliable US scores, with comparable sensitivity and specificity, suggesting that they can be variously and alternatively employed for the diagnosis of GCA and its relapses (6).

Nevertheless, some difference was assessed when our patients were distinguished according to sex: for OGUS, men displayed poorer specificity and sensitivity, even with an optimal cutoff higher than women.

Conversely, statistical analysis evidenced a lower cutoff value for HS in men, which nevertheless led to a poorer LR and a statistically significant lower specificity.

Those findings presumably mean that in men with suspected GCA, OGUS and HS are by far less specific (and OGUS less sensitive, too) than in women, presumably due to a physiological increase of IMT in men.

On the opposite, no difference was assessed for HC, whose cutoff remained the same, with identical specificity, sensitivity, and LR, in men and women: Regardless of sex and age, an HC ≥1 is strongly associated with a diagnosis of GCA.

When patients were stratified for age, no difference was evidenced for any of the scores: This, at least for HS, is in contradiction with previous findings, displaying a higher IMT in older patients, but can be explained by the reduced age range of our cohort, as well as by the high diagnostic accuracy of US, regardless of age.

When patients were stratified according to disease extent, no relevant differences were assessed for optimal cutoff, which remained the same for HC and HS. At the same time, specificity did not vary for any of the three scores, ranging from 94 to 97%; conversely, at our cutoffs, sensitivity appeared lower for HC and, particularly, HS (65%) in patients affected by cranial GCA.

In this specific subset of patients, the application of compressibility sign resulted, in our cohort, in higher sensitivity (89%) and a 100% specificity. Such findings are substantially in line with previous studies (19–21), which nevertheless did not distinguish between cranial and LV-GCA. Our findings remark that a dynamic US evaluation, comprising compression sign, is mandatory for achieving a higher sensitivity: Reduced compressibility of any segment of TA markedly increases the diagnostic value of US and should be routinely employed in a patient with suspected GCA. Further scores should therefore include compression sign and add it to the assessment of IMT and halo, thus resulting in a semiquantitative score comprehensive in all these three aspects.

Our study has some limitations: First, the relatively low numbers do not allow any definite conclusions. Second, the number of “pure” LV-GCA, is low in comparison with cranial and cranial and LV combination, thus potentially leading to an incorrect assessment of specificity and sensitivity in this subset of patients. Third, we did not evaluate subclavian (22, 23) nor vertebral arteries, which in our clinical practice are assessed only in patients with suspected Takayasu arteritis. Fourth, we employed two different US machines, although from the same factory and with comparable features. Fifth, we did not assess the echo-texture of the vessels: We suspect that in the case of subjects referred for disease relapse or with long-term disease, a chronic thickening of IMT can be misleadingly interpreted as inflammatory, instead of a fibrotic, reparatory process; hence, the inclusion of relapsing patients may be a confounder. Nevertheless, in the context of a real-life study, we could not exclude such an important subtype of patients referred to our centers.

In conclusion, all proposed scores appear feasible and reliable not only for studies or clinical trials but also in clinical practice. The high specificity assessed in all of them confirms the excellent diagnostic value of US in suspected GCA, in a clinical setting like ours which does not employ TA biopsy nor routinely requests radiological imaging procedures, such as MRI or PET, as first-line test for GCA. Despite the lack of direct comparison among OGUS, HS, and HC, the latter could be potentially preferred, as it is not influenced by age or sex.

## Data availability statement

The original contributions presented in the study are included in the article/supplementary material, further inquiries can be directed to the corresponding author.

## Ethics statement

The studies involving humans were approved by Comitato Etico Area Vasta Sudest Toscana. The studies were conducted in accordance with the local legislation and institutional requirements. The participants provided their written informed consent to participate in this study. Written informed consent was obtained from the individual(s) for the publication of any potentially identifiable images or data included in this article.

## Author contributions

EC: Conceptualization, Data curation, Methodology, Supervision, Writing – original draft. PF: Conceptualization, Data curation, Methodology, Supervision, Writing – original draft. SA: Conceptualization, Data curation, Formal analysis, Methodology, Writing – original draft. SiG: Conceptualization, Data curation, Investigation, Writing – review & editing. CB: Investigation, Supervision, Writing – review & editing. FB: Investigation, Validation, Writing – review & editing. StG: Investigation, Supervision, Writing – review & editing. MB: Investigation, Supervision, Writing – review & editing. CF: Supervision, Writing – review & editing. LC: Supervision, Validation, Writing – review & editing. BD: Supervision, Validation, Writing – original draft. BF: Supervision, Validation, Writing – review & editing.
